# Turning off the BCL-2 switch to prevent intestinal tumorigenesis

**DOI:** 10.18632/oncotarget.8757

**Published:** 2016-04-15

**Authors:** Maartje van der Heijden, Douglas J. Winton, Louis Vermeulen

**Affiliations:** Laboratory for Experimental Oncology and Radiobiology (LEXOR), Center for Experimental Molecular Medicine (CEMM), Academic Medical Center, AZ, Amsterdam, The Netherlands

**Keywords:** intestinal stem cells, chemoprevention, colorectal carcinoma, apoptosis, Bcl-2

Colorectal cancer (CRC) is one of the leading causes of cancer-related deaths worldwide. Therefore, development of effective (chemo) preventive strategies is an intense field of study. CRC is a disease that develops in the course of many years from the first oncogenic hit causing a premalignant lesion to invasive growth and ultimately metastatic disease. The oncogenic mutations that underlie the different histopathological stages have been fairly well characterized [1]. Commonly the first hit is an inactivating mutation of the *Apc* gene in intestinal stem cells (ISCs). This leads to hyperactive Wnt signaling and an evolutionary advantage of the affected stem cell over neighboring wild type ISCs, eventually giving rise to premalignant lesions with uncontrolled cellular proliferation [2]. Although the initial oncogenic hits are known and the effects on ISC population dynamics have been elucidated, the cellular adaptation process following gain of a tumorigenic mutation remains largely elusive. However, this knowledge is crucial to develop effective chemopreventive strategies for cancer in a rational manner.

Potential chemopreventive agents for CRC that received most attention are nonsteroidal anti-inflammatory drugs (NSAIDs), such as celecoxib and aspirin. In most studies these drugs have only moderate inhibiting effects on cancer development and are associated with significant side-effects upon long-term treatment. These drugs are thought to act primarily through inhibition of microenvironmental-derived, inflammation-associated signals, which renders the epithelial compartment more susceptible to malignant transformation when not opposed. In order to find novel epithelial cell specific chemopreventive targets, we aimed to uncover cell-intrinsic properties that facilitate a competitive advantage upon acquiring oncogenic mutations.

It is generally accepted that ISCs can efficiently give rise to premalignant growth upon *Apc*-loss, whereas the intestinal differentiated cell (IDC) population lacks this ability [3]. However, intestinal epithelial NF-κB activation, such as seen in colitis, greatly enlarges the pool of intestinal epithelial cells that can effectively give rise to tumors [4]. We found that high *Bcl-2* expression was one of the relatively few shared features in both scenarios, as *Bcl-2* is both an NF-κB target gene in the intestine and highly expressed in ISCs [5]. As hypothesized, we found that genetic deletion or pharmacological inhibition of BCL-2 significantly impaired tumor formation in a genetically engineered adenoma mouse model, which allows for conditional inactivation of *Apc*. Critically, BCL-2 inhibition does not affect intestinal homeostasis, yet facilitates regeneration after irradiation-induced tissue damage.

**Figure 1:**
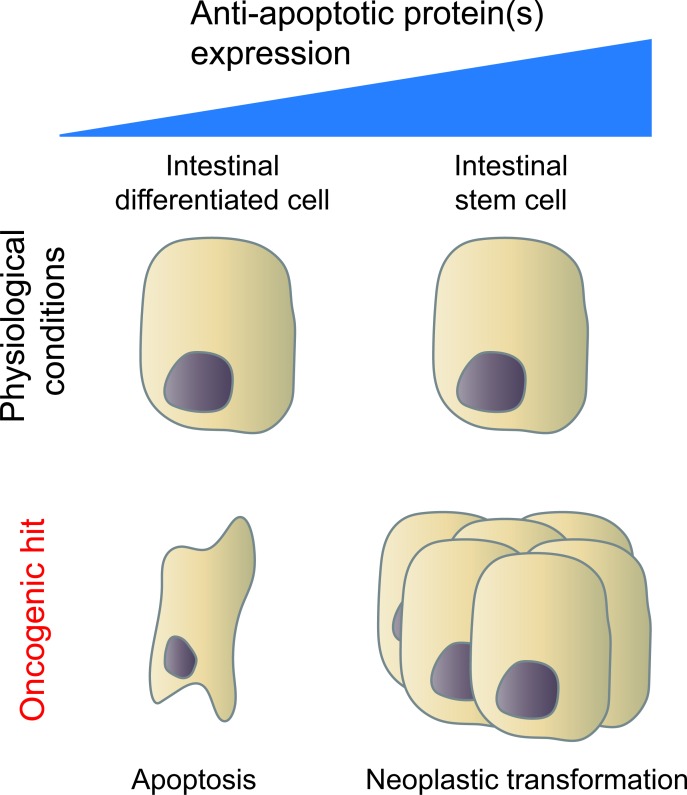
Expression of anti-apoptotic proteins facilitates neoplastic transformation in certain cell types The upper panel depicts cells with both low (e.g. intestinal differentiated cells) and high (e.g. intestinal stem cells) expression of an anti-apoptotic protein that are unaffected by oncogenic mutations in homeostasis. The lower panel (left) shows cellular apoptosis caused by stress due to an oncogenic hit in a cell with a low apoptotic threshold due to low expression of an anti-apoptotic protein. The lower panel (right) depicts neoplastic transformation of a cell that is affected by the same oncogenic mutation but has a high expression level of an anti-apoptotic protein and therefore a high apoptotic threshold.

Our data shows that there is a synthetic lethal interaction between *Apc* and *Bcl-2* in intestinal cells (Figure 1). We show that *Apc*-loss leads to elevated apoptotic priming via the intrinsic pathway, which is alleviated by BCL-2 activity in ISCs thereby preventing apoptosis in these cells. Also, increased apoptotic priming via the extrinsic apoptotic pathway has been observed upon *Apc*-loss. This latter pathway is regulated by death receptors on the outer cellular membrane that respond to microenvironmental-derived signals. Activation of oncogene *c-myc*, a Wnt target gene, represses the anti-apoptotic signal c-FLIP resulting in increased sensitivity to TRAIL and RAc [6]. Evidently, drastic alterations of generally tightly regulated signaling pathways disturb the fine balance between pro- and anti-apoptotic signals. This provides a window of opportunity to selectively target these cells that have recently accumulated oncogenic aberrations, and undergo a rewiring of their signal transduction networks, but have not yet acquired secondary and tertiary mechanisms to evade cell death.

Similar dependencies on anti-apoptotic proteins following oncogenic hits are seen in other cell types. This has especially been well-studied in the development of hematological malignancies. For example, in B-cell lymphomas *Bcl-xL* expression is crucial for cellular survival upon *myc*-induced lymphoma development, whereas *Bcl-2* is dispensable in this context [7].

Our study explains why the ISC population is more susceptible to neoplastic transformation compared to the IDCs. Unfortunately, the cells-of-origin of many cancer types are currently not well-defined, therefore identifying lineage specific survival signals might be challenging but nonetheless an attractive strategy to pursue in order to develop chemopreventive strategies for other malignancies as well.

We realize that in order to develop BCL-2 inhibition as a viable chemopreventive option for CRC in selected populations the safety profile of these agents needs to be further enhanced. Furthermore, our study focused on a mouse model of intestinal tumorigenesis and it remains to be seen if BCL-2, or other members of the BCL-2 family are equally important in human intestinal carcinogenesis. These studies are currently ongoing.
